# Supplementary material for: Development of strategic social information seeking: Implications for cumulative culture
Source: PLoS One. 2021 Aug 24;16(8):e0256605. doi: 10.1371/journal.pone.0256605 (PMC8384161; doi:10.1371/journal.pone.0256605)
Supplement: S1 Table — (DOCX) [file pone.0256605.s001.docx]

### **Supplementary information**

Prior to running each GLMM we standardized the continuous fixed effect variables (age in days and trial number) used in the model. To do this, we calculated the mean and standard deviation for the variable, then for each observed value of the variable we subtracted the mean and divided by the standard deviation. Table S1 details the model output for the standardised fixed effects. The coefficients for the other fixed effects and interactions were the same as is reported in the main text.

**Table S1.** GLMM outputs for standardised fixed effects

| Model | Standardised Fixed effects | *b* | SE | *z* | *p* |
| --- | --- | --- | --- | --- | --- |
| Information seeking | Age | 1.81 | 0.29 | 6.32 | <.001 |
|  | Trial number | 0.35 | 0.12 | 2.93 | .003 |
|  | Age*Trial number | 0.26 | 0.13 | −2.04 | .041 |
| Information use | Age | 1.13 | 0.18 | 6.26 | <.001 |
|  | Age*Demonstration outcome | 0.32 | 0.15 | 2.09 | .037 |
