# Supplementary material for: Development of strategic social information seeking: Implications for cumulative culture
Source: PLoS One. 2021 Aug 24;16(8):e0256605. doi: 10.1371/journal.pone.0256605 (PMC8384161; doi:10.1371/journal.pone.0256605)
Supplement: Attachment — Submitted filename: Response to Reviewers.docx [file pone.0256605.s002.docx]

Philosophy
Faculty of Arts and Humanities
University of Stirling
FK9 4LA
United Kingdom

July 30, 2021

Dear Mark Nielsen, Ph.D.,

Thank you for the feedback and comments from yourself and the two reviewers. We found these comments to be constructive and extremely helpful in improving the clarity of the manuscript. We are therefore pleased to offer our revised manuscript. We have provided our responses to the individual review comments in blue in the Response to Reviewers file.

Thank you for the opportunity to make these revisions.

Yours sincerely,

Kirsten H. Blakey, Eva Rafetseder, Mark Atkinson, Elizabeth Renner, Fía Cowan-Forsythe, Shivani J. Sati, & Christine A. Caldwell

Reviewers' comments:

Reviewer's Responses to Questions

**Comments to the Author**

1. Is the manuscript technically sound, and do the data support the conclusions?

The manuscript must describe a technically sound piece of scientific research with data that supports the conclusions. Experiments must have been conducted rigorously, with appropriate controls, replication, and sample sizes. The conclusions must be drawn appropriately based on the data presented.

Reviewer #1: Yes

Reviewer #2: Partly

2. Has the statistical analysis been performed appropriately and rigorously?

Reviewer #1: Yes

Reviewer #2: Yes

3. Have the authors made all data underlying the findings in their manuscript fully available?

The [PLOS Data policy](https://eur03.safelinks.protection.outlook.com/?url=http%3A%2F%2Fwww.plosone.org%2Fstatic%2Fpolicies.action%23sharing&data=04%7C01%7Ck.h.blakey1%40stir.ac.uk%7C48d343ab754f42b9ad9408d90fcf44c0%7C4e8d09f7cc794ccb9149a4238dd17422%7C0%7C0%7C637558205888453462%7CUnknown%7CTWFpbGZsb3d8eyJWIjoiMC4wLjAwMDAiLCJQIjoiV2luMzIiLCJBTiI6Ik1haWwiLCJXVCI6Mn0%3D%7C1000&sdata=zjH1iyPC8InnZLE2cdeM8TAtS6DcRKJYPBHh3ijzgRI%3D&reserved=0) requires authors to make all data underlying the findings described in their manuscript fully available without restriction, with rare exception (please refer to the Data Availability Statement in the manuscript PDF file). The data should be provided as part of the manuscript or its supporting information, or deposited to a public repository. For example, in addition to summary statistics, the data points behind means, medians and variance measures should be available. If there are restrictions on publicly sharing data—e.g. participant privacy or use of data from a third party—those must be specified.

Reviewer #1: Yes

Reviewer #2: Yes

4. Is the manuscript presented in an intelligible fashion and written in standard English?

PLOS ONE does not copyedit accepted manuscripts, so the language in submitted articles must be clear, correct, and unambiguous. Any typographical or grammatical errors should be corrected at revision, so please note any specific errors here.

Reviewer #1: Yes

Reviewer #2: Yes

5. Review Comments to the Author

Please use the space provided to explain your answers to the questions above. You may also include additional comments for the author, including concerns about dual publication, research ethics, or publication ethics. (Please upload your review as an attachment if it exceeds 20,000 characters)

**Reviewer #1:** The manuscript investigates the development of strategic social information seeking in children aged 2-8. The authors report an experiment in which implicit SLSs would not be beneficial for the learner and, instead, metacognition would be necessary to perform well in the experiment. The authors consider that metacognition could explain why cumulative culture is present in humans but not in other animals. They provide a comprehensive literature review and rationale for their hypothesis. The experiment is well designed, and the statistical analysis are strong. In conclusion, this manuscript is a very valuable addition to the literature, and I recommend its publication. I only have a few minor comments and suggestions:

Line 74: Heyes’ dual process is introduced here. The first part of the process should be clearly stated here and Heyes should also be mentioned. She is mentioned later when explaining the second part of the dual process.

The manuscript has been amended to include this suggestion.

Lines 98-107: these lines are very interesting. I was wondering to what extent implicit SLSs are domain-general or domain-specific. Although the literature has found some evidence of copying of high prestige/older individuals over low prestige/younger individuals, this does not seem to be always the most adaptive strategy. For example, an old individual might prefer to learn IT from a younger individual than from an older one. Could implicit SLSs alone account for this domain-specificity or metacognition is necessary to fine-tune SLSs to each domain?

Agreed, while strategies for copying high prestige/older individuals over low prestige/younger individuals are not *always* the most adaptive, they are the most adaptive strategies in the broadest range of contexts. Therefore, implicit SLS could be considered to be domain-general, while domain-specific cases of social learning such as learning IT would be beyond their scope and likely require explicitly metacognitive SLSs. Indeed, Heyes sets out metacognitive SLSs (giving examples of copying digital natives or boat builders with the biggest fleet) as being domain-specific as success in such cases requires some degree of recognition of the problem one faces, what information is required to solve the problem, and who can provide the relevant information. In the interests of keeping the introduction concise and focussed we have not specifically mentioned this, however if the reviewer feels that this should be included then we would be happy to do so.

Lines 215-256. I understand you only have one hypothesis: there will be “an age-related transition an age-related transition from the use of heuristic mode-based biases to reasoning-based choices driven by the value of the information”. I think you could make this stand out more by stating the hypothesis in a separate paragraph.

This is now a separate paragraph.

Statistical analysis
Line 354: I think a definition of appropriate information seeking and appropriate information use is needed here. You do this later in the results section.

As suggested, we have included these definitions.

Lines 358-359: A sentence explaining why random effects where necessary for your experimental design would be beneficial for the reader.

We have added a sentence to explain this into the statistical analysis section.

Results
I would like to see some measure of effect size or to have a way to compare the strength of the different predictors in the regression models (e.g. standardized coefficients, using information criteria to compare the fit of different models taking in and out specific predictors). This could be reported in a table as supplementary materials.

We have not added a comparison of the fit of different models taking in and out specific predictors as there are varying ways to build such models (Meteyard & Davies, 2020). In building our models we have ensured that all fixed effects were sum coded, centred or scaled as appropriate, and aimed to keep the random effects structure ‘maximal’ where possible in line with Barr et al. (2013). In order to report models consistently with other recent work (e.g., Atkinson et al., 2020; Renner et al., 2021; Wilks et al., 2021) the manuscript reports the coefficients for all fixed effects included in the model and those for the significant interactions (as well as post hoc analyses). For each model we have added a sentence to explain that the models that are reported were compared to (and are significantly better fits than) their null equivalents.

We have included a table in supplementary information which details the standardised coefficients for the continuous variables in each model (age and trial number) and the significant interactions they are involved in. As it is not appropriate to standardise categorical variables that have been sum coded these are reported only in the main text.

We recognise that there are different ways that researchers might want to analyse the data, therefore we have made the data and analysis code available in an OSF repository.

I was wondering whether the section of information seeking errors is necessary. The results are the same as for information seeking: gender congruency is a predictor of information seeking, females prefer female models, but males do not show a preference for either males or females.

Thank you for highlighting the redundancy here, we agree with this assessment and have now removed the information seeking errors section.

The symbol of chi squared does not look right: lines 436, 438, 443, 444, 447.

This section has now been removed as per the suggestion above.

Line 500: ordinal (from ordinal package) needs italic.

This has now been corrected.

499-512: Did you use random effects for the ordinal models? There is no mention of this here.

No random effects were included in the ordinal models.

Figures 2, 3, and 5. I would like to see the numbers for the proportions for the different categories as this is shown in figures 4, 6, and 7.

As suggested, we have added these numbers to the figures.

Overall statement
I really like this manuscript. Congratulations to the authors for a very valuable contribution to the field of Social Learning.

**Reviewer #2:** This is a well-written paper on the development of “explicitly metacognitive” social learning strategies and their implications for the evolution of cumulative culture. The study was designed to investigate “when children develop the ability to seek out social information using explicitly metacognitive SLSs” (l. 215/216). The strong points of the study include its rather large sample size, the data availability, the use and clear description of appropriate data analysis methods, and the clear presentation and visualisation of the results. The introduction is long, but an interesting and informative read and demonstrates that the authors know the literature well and have grounded their research question well into theory. My comments relate mostly to the introduction (some concepts could be defined better), the study design (it is currently unclear why the chosen task investigates social information seeking rather than information seeking in general), and the discussion (some conclusions are not unequivocally supported by the data). I am especially unclear to what extent the task used in this study tests for the strategic use of social information – and this should be made much clearer. Many of my comments relate to this issue. However, I think these issues could be addressed in a minor revision. I added my comments point-by-point and chronologically below, with a few minor points at the end.

Introduction, l. 77/78: “Explicitly metacognitive SLSs are defined as consciously represented and reportable rules.” It would be great if this sentence could be specified to explain rules regarding what. Maybe an example could be added to help explain what explicitly metacognitive SLSs are.

We have added a clarification in this sentence to make clear that these rules reflect an explicit awareness and understanding of the reasoning-based learning strategies being employed. As suggested, we have also added an example.

Introduction, l. 80/81: “Such strategies involve the use of theory of mind and metacognitive processes”. Here, explicitly metacognitive SLSs are described as involving metacognitive processes, which is somewhat tautological. It remains unclear what is meant by metacognitive processes. I suggest replacing the wording “metacognitive processes” here with something more explanatory.

We agree that this is too vague, we have therefore added clarification to this sentence.

Introduction, l. 82-85: “As such, explicitly metacognitive SLSs afford learners the capacity to reason about what information is required and recognize the potential value to themselves of information that can be provided by others.” I find this description slightly contradictory – or at least not fully consistent – with the information provided further above in the introduction that SLSs can be described as rules, which assumingly help the individual e.g., to identify whom to learn from. Here, SLS are described as allowing the individual to reason about what information is required in the first place. This seems to me to be a different kind of demand, both in time (first I need to identify which information is needed, then I can use a rule that I can use to get that kind of information in my social environment) and in terms of the cognitive demands. It seems that there is a conflation between different demands: recognizing what information is required and using social learning strategies to help select the appropriate social information. Thus, I suggest describing in an even clearer way what exactly explicitly metacognitive SLS are and what they allow a learner to do (and also what it is not).

We have edited this section to give a clearer outline of explicitly metacognitive SLSs, and in line with a previous suggestion.

Introduction: It should also be clarified in what way explicitly metacognitive rules rely on language. The manuscript mentions that this type of SLSs consist of reportable rules – does this mean verbally reportable?

We have added a clarification to our definition of explicitly metacognitive SLSs to make clear that these rules may be reportable for verbal individuals. Though these strategies are proposed to be unique to humans, language would not necessarily be required for an individual to demonstrate the capacity for explicitly metacognitive SLSs.

Introduction, l. 108-110: “While there is evidence of implicit SLSs, based on heuristic biases, in both young children and animals, in our view, there is as yet no solid evidence of explicitly metacognitive SLSs in either population.” It could be added here what kind of evidence would be required to prove the existence of explicitly metacognitive SLSs in animals, especially because above it was mentioned that these SLSs are reportable rules – if this means verbally reportable, then the existence of such SLSs would a priori not be possible in animals. This should be clarified in the manuscript.

The capacity to verbalise a strategy is not in itself the distinguishing feature between the strategies, because as the reviewer notes, this would exclude all non-humans by default. We hope that the clarifications now included in response to the previous comment address this point.

Introduction, l. 110-115: “adult humans are (with good reason) assumed to be able to use social information in an explicitly metacognitive manner […] For example, it is routine for human adults to actively seek out models of social behaviour, using their understanding of others’ intentions relative to their own to strategically select and use the most relevant social information […]. A critical question, then, is how children’s use of social information develops with age, particularly in relation to their explicit understanding of the value of social information” The use of explicitly metacognitive SLSs is here exemplified by knowing which social models to seek out and using others’ intentions to select information. Then the question is asked how these abilities develop in children. However, the task used in the current study does not require a selection between social models or reading others’ intention – instead, as several comments of mine show, it does not really become clear in what way the task used here is social. It seems that children just need to focus on the boxes shown in the video stills (which box looks like the one I have in front of me?) and that the demonstrators can be ignored. The value of information lies in the type of boxes, not in the models themselves. In short, I find that there is a gap between the theoretical description of explicitly metacognitive SLSs and what they entail on the one hand and how this is implemented in the current task. This information should be added to allow the reader to follow the argument.
Introduction, l. 134-136: “in the current study we were particularly interested in determining when children develop the ability to seek out social information based on an understanding of the value of that information.” It should be explained why the inclusion of “social” is warranted here. It seems that the task used just tested the ability to seek out information based on an understand of the value of this information.

Adults do not only use understanding of others’ intentions they also use their understanding of others’ knowledge or access to relevant information to seek out appropriate information, we have expanded this sentence to include these additional examples to clarify our meaning.

It is true that children should have focused on the boxes rather than the characteristics of the demonstrators, however, the information about the box can only be obtained via the social demonstrator. The task in the current study did require children to make a selection between social models, however, the difference between this study and what has been investigated in previous studies is that only one of the social models here can provide information that is relevant for the problem the child is faced with. This affords us the opportunity to examine whether children’s selections are driven by a recognition of what information is required to solve their problem and where (which model) they can get that information, or whether they have not recognised the relevance of the information that can be provided by the models and instead make their selections based on heuristic model-based biases. We have added an additional section to the introduction for clarification of these points.

Introduction, l. 183-188: “While it is useful to know how children use social information and who children prefer to learn from, we argue that this is not sufficient to determine the cognitive mechanisms that children are employing during social learning. Children’s ability and proclivity to seek out relevant social information has not yet been adequately addressed. That is, nothing in the SLSs or selective trust literature has examined children’s ability to select valuable social information on the basis of its relevance for solving a specific problem.” Could this question be rephrased to “when do children become able to find the correct solution to a problem?” Again, as mentioned above, I am struggling to find the reasoning for including “social” here. Also, would the answer to the question not also depend on the specific problem and the experience that children have related to the problem? This should be discussed.

We hope that the clarifications now included in other parts of the introduction have addressed this point.

Introduction, l. 244-248: “Appropriate use of social information (following appropriate information seeking) could further help to distinguish between learners with some level of metacognitive understanding, and those reliant on implicit rules for what, when, and whom to copy. That is, understanding the relevance of the acquired information would be expected to also result in more appropriate use of that information.” I would suggest adding a bit more information on how exactly the use of social information can provide additional insights into the use of social learning strategies over and above the seeking out of relevant information. This becomes a bit clearer when reading the discussion of the paper, but is not very clear at the introduction. Adding an example to illustrate this point would be helpful here to let the reader follow the argument. In other words, how exactly can finding out how children use social information tell us about the nature of their social learning processes in addition to finding out how children seek the information?

Thank you for highlighting the ambiguity here. In addition to the responses to previous comments that are related to this, we have added further explanation to this section.

Methods, 376-379: “Appropriate information seeking was measured by the proportion of trials in which children selected the target video. This required children to reason about the information they needed to solve a problem (unlock their box) and which of the demonstrators could provide that information (which of the demonstrators were facing the same problem).” The second sentence suggests that children would have to attend to two kinds of sources of information to make the correct choice: the box depicted in the video and the demonstrator. It is unclear to me why attention to the demonstrator is needed – children could just select the video that shows the same problem as theirs and disregard the demonstrator. Indeed, this is the point of the study: “If children were reasoning appropriately, they should have disregarded demonstrator characteristics” (l. 379/380). Therefore, this second sentence should be rephrased as appropriate information seeking did not require reasoning about which demonstrator could provide information.

It is not accurate to say that appropriate information seeking did not require reasoning about which demonstrator could provide information, rather it is the characteristics of the demonstrators that was not relevant to participants choice. That is, the information could not be obtained without recognition of which video could provide relevant information and therefore which demonstrator could provide that information. Basing a choice on the age or gender of the demonstrator without considering whether or not they could provide relevant information would be inappropriate. We have edited the second section highlighted in this comment to make this clearer.

Discussion, l. 527-530: “Specifically, we were interested in identifying when children develop the ability to seek out information based on a reasoned understanding of what information is required to solve a particular problem and who could provide it (explicitly metacognitive SLSs).” Similar to the previous comment, I am questioning whether being successful in the task used in the current study required both attending to what information is required and who is able to provide it. It seems that attending to what information is required was sufficient. If both information and demonstrator had to be attended to, the design should have been different, e.g., there being two or more demonstrators with the correct box, but only one of them being able to provide the information for whatever reason (e.g., if children wouldn’t be able to watch the video, but just saw the still, and then had to rely on a verbal report (in a different video) by the demonstrators, but for some reason only one demonstrator could provide that report (e.g., the others cannot speak or cannot be heard). I suggest rephrasing this sentence accordingly or to explain in more detail why attention to both the type of box and the demonstrator is required.

We recognise now that our phrasing here may have been slightly misleading. We are really only talking about one decision, but this decision relates to attending to the available social information (in the form of observation of the demonstration). We have reworded this sentence to make this less ambiguous.

In addition, also referring to the same sentence, the discussion section fails to answer this question. While it is discussed that children’s performance in the task increases with age, no answer is given to when the ability to seek out information based on its value emerges. As performance in all age groups was above chance levels, I assume the answer here would be that this ability emerges very early in life, before 3 years of age. This should be added and discussed.

The discussion has been amended to ensure that this question is answered as clearly as the data allow.

Results: I am wondering whether the authors have investigated whether there is a trial effect on whether or not mistakes are made, i.e., whether the unsuccessful trials tended to be the first trials (especially because there did not seem to be a warm-up or a check to see whether children had understood what to do). I am wondering whether errors occurred because children initially did not quite understand what to do and then fell back on an implicit SLS or there was no trial effect, indicating that some of the errors were made not because the children did not understand the task, but because a pull of an implicit SLS was too strong.

Thank you for making this suggestion. We re-ran the information seeking analysis to include trial number as a fixed effect. The results are now included in the Information seeking section of the results. The pattern of errors appears to be consistent with your suggestion as the analysis revealed a main effect of trial number indicating that information seeking improved with task experience. However, an interaction between trial number and age indicated that this trial effect was restricted to older children. Therefore, consistent with our interpretation, the lack of a trial effect in younger children suggests that their errors were the result of relying on implicit SLSs (in this case model-based biases for gender congruent demonstrators) due to not having the capacity for explicitly metacognitive SLSs (i.e., not recognising which of the demonstrators could provide information relevant to solving the problem). Whereas older children were capable of solving the task but may have taken a trial or two to figure out what was expected from them.

Discussion, l. 535/536: “We found that the proportion of trials in which children selected the target demonstration video increased with age. This corresponded to our prediction that older children would exhibit more appropriate social information seeking than younger children.” Again, more explanation is needed as to why the behaviour investigated in this study can be labelled social information seeking. What is the social here, especially because a correct response to the task would imply disregarding social information (i.e., information about demonstrator characteristics)?

We hope that the clarifications now included in other parts of the manuscript have addressed this point.

Discussion, l. 544-552: “Thus, we suggest that older children understood the potential value to themselves of the information that the target demonstrator could provide having employed an explicitly metacognitive SLS. By contrast, we postulate that younger children’s target video selections were less likely to have been driven by reasoned understanding of the information they needed and the potential value of the target demonstration video. Therefore, when selecting a video, younger children might have instead relied on less cognitively demanding implicit SLSs such as model-based biases, or a bias for selecting models whose box matched their own box (without appreciating the value of the information).” More information needs to be provided (already in the introduction, and possible again here) why choosing the correct video requires an “explicitly metacognitive” understanding of the information that was needed. What about alternative explanations, e.g., that simply recognizing the box that is in front of oneself in one of the video captures? Second, I might be misunderstanding what children needed to do in the task (and if so, this should be made clearer) but did they not have to select the video with the model whose box matched their own box? I am struggling to follow the argument that young children might have had a bias for selecting models whose box matched their own. More information should be added here to allow the reader to follow the argument. In general, I think that the introduction could include more specific information on what specific demands the task poses and why.

While it is true that simply recognising that one’s own box matches the one in one of the pictures would lead to successful selection of the target video, it is unlikely that this would also result in success in the information use part of the task. This is where examining information seeking and information use in conjunction with one another offers a key insight into the SLSs being employed. For instance, if children have selected the target video simply by matching the boxes, then they may not know what to do with the information once they have received it leading to poorer selections of the target key. Whereas if children have selected the target video based on an understanding of why that information is relevant, we would expect them to also know what to do with that information, thus they would be expected to select the target key more often. We have added an additional explanation of this to the introduction and to the discussion.

Discussion, l. 563-565: “Results showed that children were more likely to select target demonstrators who were the same gender as themselves, therefore showing a gender congruence effect.” I am not convinced that the data support this conclusion. The gender congruence effect was only observed in girls, and so does not apply to the entire sample. Thus, the conclusion should be adjusted accordingly.

We have now changed this part of the discussion to reflect the gender difference with regard to this effect. We have also added clarifications related to the 3 way interaction found between participant-target demonstrator gender congruence, gender, and target demonstrator age, because male children did show a gender congruence effect when the target demonstrator was an adult though they did not show an overall effect.

The following point as several comments:

Discussion, l. 568-570: “Therefore, it seems that even when it was apparent – from salient visual cues – which demonstrator could provide relevant information”. Related to a previous comment, I find this phrasing misleading, as this suggests that solving the task needs attention to the demonstrator at all or asking the question “which demonstrator provides the information I need”. However, the visual cues that are mentioned here relate to the box only, not to the demonstrator. I suggest clarifying this and similar sentences throughout the manuscript.

Another issue is that the relevant information lies outside of the demonstrator (box) and not within them. That is, the task could also be solved if the videos only showed the boxes and them being opened with the demonstrator and their hands digitally removed. Therefore, also related to a previous comment, the task is not inherently social it seems to me, or at least it should be better explained why it should be regarded as one.

Yes, the visual cues here refer to the box, however as outlined above, the information about the box is obtained by recognising which of the demonstrators has access to that information and choosing to watch the video of that demonstrator. We have tried to make this clearer throughout the manuscript.

Adding the demonstrator just adds the potentially distracting information on age and gender. The study could also have used a different design, in which the relevant information is “inside” the demonstrator, e.g., via a knowledge state, and could contrast with heuristic biases. Why was it chosen to have the value of information in the separate object rather than keeping it attached to the demonstrator?

We accept that there are alternative ways in which we could have run the study such that several demonstrators were observed attempting to open particular boxes (with the demonstration itself obscured), and then (with boxes removed) the child could be asked to select which of them to ask about which key they should use. However, while this method would test the same question, there would be considerable additional cognitive demands (e.g., memory). Therefore, in this study, we chose to make it visually salient to children which of the demonstrators had access to the relevant information. In the interests of brevity, we have not mentioned the alternative method in the manuscript, however, we would be happy to propose this as a more “demonstrator-internal” method of testing the same question if the reviewer feels that this would be a worthwhile addition.

The fact that there are two potential sources of information (object and demonstrator) puts a (possibly for the current research question unnecessary) demand on attentional resources on children. Therefore, it could be possible that younger children were overwhelmed by the richness of the information provided in the videos and that this could have led them to fall back on simpler strategies rather than them being unable to seek out the correct information per se. This potential limitation should be discussed.

We do not believe that including the demonstrators would have increased the attentional demands of the task, as if children have the capacity for explicitly metacognitive SLSs then understanding what information is required to solve the problem and where that information can be obtained (i.e., which of the demonstrators has access to that information) would likely override implicit SLSs. If it was the case that children were capable of explicitly metacognitive SLSs but were overwhelmed by the available information in video stills, then those children who did select and watch the target video would have been expected to be able to use the information once it had been provided. However, information use (selection of the target key) in younger children was found to be no different to chance, thus they appear not to know what to do with the relevant information when they did receive it. This suggests that they were relying on implicit SLSs because they did not have the capacity for explicitly metacognitive SLSs. We have added further discussion on this to the discussion section.

Discussion, l. 627-630: “However, as we used age as a proxy for cognitive development and developmental trajectories of other cognitive capacities overlap with that of explicit metacognition, we cannot say for certain which cognitive developments might be implicated in the emergence of the capacity for explicitly metacognitive SLSs.” It could be added here that future studies would need to investigate whether individual differences in the use of explicitly metacognitive SLSs correlate with differences on other tasks measuring these other putative cognitive developments.

Thank you for this suggestion, we have now added a sentence to this effect.

Discussion, l. 653: The discussion mentions that children in the current study were able to actively seek out appropriate social information. As mentioned above, an explanation is needed why the information provided in the videos is social.

Information should be provided on the question whether it can be ruled out that the younger children failed to understand the task, e.g., that they could only choose one key, and also whether the younger children were motivated enough (I understand they could not keep the rubber duck?). Did the younger children understand at all that they were in need of more information?

It is possible that younger children did not understand that they were in need of information, however, this itself would be an indication that they were not using explicitly metacognitive SLSs. Older children overwhelmingly demonstrated that they had recognised this. Children were not told in advance what was inside the boxes, and all voluntarily took part in the task and had the option to stop at any time. They received a sticker as a reward at the end although the reviewer is correct that they were not permitted to keep the duck. Nonetheless, we do believe that children were certainly motivated to open the boxes. Only two children were excluded from the sample as a result of wishing to stop and/or not enjoying the task. All remaining children continued to the end and appeared to be enjoying the experience.

Discussion, l. 667-671: “explicitly metacognitive SLSs are experience-dependent and develop relatively late in children. The relatively late emergence of the capacity to use reasoning-based, or explicitly metacognitive, SLSs suggests that this may be a cognitive mechanism that is unique to humans and is unlikely to be observed in animals”. The argument that SLSs are experience-dependent and that they may rely on uniquely human cognitive abilities are two different, but not mutually exclusive, possibilities. This should be made a bit clearer: what is it that younger children are likely to lack? Certain cognitive mechanisms or general experience from the social and physical world?

The reviewer is correct that these are two separate – though related – points. The first is that the late emergence is consistent with the experience-dependence which the proposal asserts. The second is that late development (but not necessarily the experience dependence) is consistent with the idea that this may be unique to humans, in particular this point hinges on the assumption that the late development is an indication that it depends on certain other cognitive developments which are also unique to humans. We have reworded this section to make this clearer.

Minor points:
- Introduction, l. 40-43: “Seeking out relevant information from appropriate social sources is ubiquitous in human adults. Human adults may therefore demonstrate key differences in the way they seek, attend to, and use social information compared to children and non-human animals (henceforth animals).” I would suggest deleting “therefore”, as the suggestion described in the second sentence does not follow logically from the first sentence.

This point has now been addressed.

- Introduction, l. 156: “Similar to the selective preferences in children’s proclivity to copy and consistent” – please doublecheck this sentence, it seems as if “consistent” should be a verb or if a verb is missing.

This sentence has now been reworded.

- Methods, l. 318: “experimenter one” – change to “experimenter 1”
- Methods, l. 358: “cantered” – change to “centered”
- Results, l. 434: “demonstrators” children” – remove apostrophe?

These changes have been made.
